# *Drosophila* innate immunity: regional and functional specialization of prophenoloxidases

**DOI:** 10.1186/s12915-015-0193-6

**Published:** 2015-10-01

**Authors:** Jan P. Dudzic, Shu Kondo, Ryu Ueda, Casey M. Bergman, Bruno Lemaitre

**Affiliations:** Global Health Institute, School of Life Sciences, Ecole Polytechnique Fédérale de Lausanne (EPFL), Station 19, 1015 Lausanne, Switzerland; Invertebrate Genetics Laboratory, Genetic Strains Research Center, National Institute of Genetics, Mishima, 411-8540 Japan; Faculty of Life Sciences, University of Manchester, Michael Smith Building, Oxford Road, Manchester, M13 9PT UK

**Keywords:** *Drosophila*, Prophenoloxidase, Melanization, Gene family, Immunity, Duplication

## Abstract

**Background:**

The diversification of immune systems during evolution involves the expansion of particular gene families in given phyla. A better understanding of the metazoan immune system requires an analysis of the logic underlying such immune gene amplification. This analysis is now within reach due to the ease with which we can generate multiple mutations in an organism. In this paper, we analyze the contribution of the three *Drosophila* prophenoloxidases (PPOs) to host defense by generating single, double and triple mutants. PPOs are enzymes that catalyze the production of melanin at the site of infection and around parasites. They are the rate-limiting enzymes that contribute to the melanization reaction, a major immune mechanism of arthropods. The number of PPO-encoding genes is variable among insects, ranging from one in the bee to ten in the mosquito.

**Results:**

By analyzing mutations alone and in combination, we ascribe a specific function to each of the three PPOs of *Drosophila*. Our study confirms that two PPOs produced by crystal cells, PPO1 and PPO2, contribute to the bulk of melanization in the hemolymph, upon septic or clean injury. In contrast, PPO3, a PPO restricted to the *D. melanogaster* group, is expressed in lamellocytes and contributes to melanization during the encapsulation process. Interestingly, another overlapping set of PPOs, PPO2 and PPO3, achieve melanization of the capsule upon parasitoid wasp infection.

**Conclusions:**

The use of single or combined mutations allowed us to show that each PPO mutant has a specific phenotype, and that knocking out two of three genes is required to abolish fully a particular function. Thus, *Drosophila* PPOs have partially overlapping functions to optimize melanization in at least two conditions: following injury or during encapsulation. Since PPO3 is restricted to the *D. melanogaster* group, this suggests that production of PPO by lamellocytes emerged as a recent defense mechanism against parasitoid wasps*.* We conclude that differences in spatial localization, immediate or late availability, and mode of activation underlie the functional diversification of the three *Drosophila* PPOs, with each of them having non-redundant but overlapping functions.

**Electronic supplementary material:**

The online version of this article (doi:10.1186/s12915-015-0193-6) contains supplementary material, which is available to authorized users.

## Background

The constant interactions of infectious microbes with their hosts explain the emergence of complex immune systems. Comparative immunology provides one of the best approaches to understanding the logic of metazoan host defense mechanisms and their diversification throughout evolution. This approach reveals that despite the apparently extreme diversity of immune reactions, similar mechanisms are used across the animal kingdom to cope with microbes. Phagocytosis, epithelial production of antimicrobial peptides, sequestration of iron or mucus barriers are found in many organisms, indicating either an ancient origin or their recurrent emergence by convergent evolution. Other immune modules are specific to a limited number of organisms. Emergence, loss and diversification of these immune modules are thought to reflect the evolutionary trajectory of metazoan lineages facing various selective pressures from pathogens. Immune diversification has often involved the expansion of particular gene families. For instance, the number of genes encoding Toll-like receptors, peptidoglycan recognition proteins and C-type lectins markedly varies among phyla [1]. A better understanding of the metazoan immune system requires an analysis of the rules underlying such immune gene amplification. The recently developed CRISPR/Cas9 genome editing approach offers a new strategy for generating mutations in a quasi-systematic manner. This allows, for the first time, the question of gene family diversification to be tackled, by generating mutations in single and multiple genes belonging to the same family. We previously reported the functional analysis of two of the three *Drosophila* prophenoloxidases (PPOs), PPO1 and PPO2, using single- and double-mutant analysis [2]. We subsequently realized that the *PPO1,PPO2* double-mutant stock used in our previous study carries a mutation in the *PPO3* gene. Here, we investigate the function of *PPO3*, and perform a single-, double- and triple-mutant analysis of the three *Drosophila* PPOs. Our study extends our previous results and attributes specific and complementary functions to each of the three PPOs, providing an insight into how this protein family has evolved in *Drosophila*.

Melanization is a major immune module found in arthropods but not in vertebrates [3, 4]. It involves the rapid synthesis of a black pigment named melanin, at the site of infection or injury. A key enzyme in melanin biosynthesis is phenoloxidase (PO), which catalyzes the oxidation of phenols to quinones, which subsequently polymerize into melanin. PO is usually synthesized as a zymogen called prophenoloxidase (PPO), which is cleaved to generate the active PO enzyme upon activation of a proteolytic cascade. Several roles have been ascribed to the melanization reaction in insects [5–8]. PO activity contributes to wound healing by forming a scab at the epithelial injury site. By-products of PO activity are reactive oxygen species, which are thought to contribute to the killing of microbes and pathogens. Finally, melanization participates in the encapsulation reaction against parasites. Deposition of melanin on the parasite forms a physical barrier, allowing the localized and confined production of toxic compounds while ensuring the protection of the host. Melanization is clearly an arthropod-specific mechanism, but its functions in providing a barrier and generating microbicidal reactive oxygen species are usually carried out by other mechanisms in other species.

The *Drosophila melanogaster* genome contains three *PPO* genes, all on the second chromosome [2]. PPO1 and PPO2 are produced in specialized hemocytes (blood cells), called crystal cells. Crystal cells represent 5 % of the hemocyte population in larvae [9, 10]. Upon injury, they rupture and release PPOs into the hemolymph (the insect blood), where they are activated by a cascade of serine proteases (SPs) [11]. Using null mutations in *PPO1* and *PPO2*, we recently showed that PPO1 and PPO2 are responsible for all the PO activity in the hemolymph [2]. While PPO1 is involved in the rapid early delivery of PO activity, PPO2 present in the crystals of crystal cells provides a storage form, which can be deployed in a later phase [2]. This and other studies also revealed an important role for PPO1 and PPO2 in survival after infection with Gram-positive bacteria and fungi, underlining the importance of melanization in insect host defense [4, 12–14]. However, previous work left open the question of the function of the third PPO, PPO3, which could not be addressed because no mutant was available. The expression pattern of *PPO3* was not clear either. Some reports suggested that *PPO3* is expressed in crystal cells [15, 16], while others proposed it is expressed in lamellocytes [17, 18]. Lamellocytes are a type of larval hemocytes that are induced upon injury or wasp infection and play a key role in the encapsulation of foreign bodies and parasites [19]. Interestingly, while PPO1 and PPO2 require proteolytic cleavage to be activated, PPO3 is thought to be produced in an active form, although a putative cleavage site is present [18, 20].

The starting point of the present study came with the serendipitous observation that the *PPO1*^*Δ*^*,PPO2*^*Δ*^ double mutant that we previously used to analyze the role of PPO1 and PPO2 [2], also carries a cryptic null deletion in *PPO3*. This raised the possibility that the phenotype initially attributed to PPO1 and/or PPO2 could be caused by the absence of PPO3. To clarify this question, we separated the *PPO3* mutation from the *PPO1*^*Δ*^ and *PPO2*^*Δ*^ mutations and additionally generated an independent *PPO3* mutant using the CRISPR/Cas9 approach [21]. Using single, double and triple mutants of the three *PPO* genes, we confirm that PPO1 and PPO2 are the only source of hemolymphatic PO upon septic injury. Our study also reveals a role for PPO3 in the encapsulation process, in association with PPO2.

## Results

### Identification of a cryptic *PPO3* deletion in the *PPO1*^*Δ*^,*PPO2*^*Δ*^ double-mutant stock

**Fig. 1:**
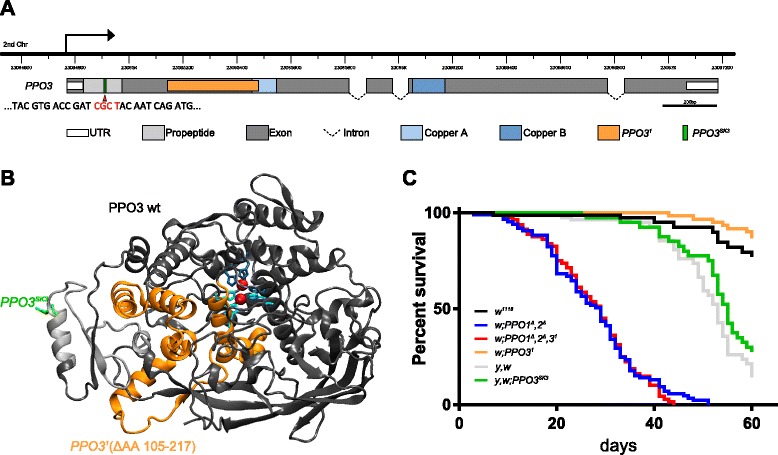
Molecular characterization of two novel *PPO3* mutations. **a** Schematic representation of the *PPO3* gene locus and *PPO3* coding sequence. The gene map was adapted from FlyBase. The *PPO3* ^*1*^ mutation was present in the *PPO1* ^*Δ*^ *,2* ^*Δ*^ double-mutant stock while the *PPO3* ^*SK3*^ mutation was generated by CRISPR/Cas9. Shown are the protein domains of PPO3 and a partial sequence where the deleted nucleotides are marked in *red*. **b** Structural modeling shows that the *PPO3* ^*1*^ mutation (*orange portion*) deletes most of the catalytic pocket, including copper-coordinating residues of the copper-binding domain A (*light blue*). Protein domains are colored as in (**a**). **c** Lifespan analysis of unchallenged flies reveals an increase in mortality rate of *PPO1* ^*Δ*^ *,2* ^*Δ*^ and *PPO1* ^*Δ*^ *,2* ^*Δ*^ *,3* ^*1*^ mutant flies but not *PPO3* ^*1*^ mutants. Each survival curve corresponds to three independent experiments with 20 flies each. *Chr* chromosome, *UTR* untranslated region, *wt* wild type

By testing primers for the *PPO3* gene, we serendipitously discovered the presence of a deletion of 336 bp, removing 112 (residues 105–217) of the 683 amino acids of the PPO3 protein (Fig. 1a). This deletion is referred to as *PPO3*^*1*^, and we use parentheses around this mutant to refer to previous results where this mutant was present in a cryptic state. As the deletion does not change the reading frame, a residual protein is expected to be produced, lacking part of a conserved copper-binding domain found in all PPOs (Fig. 1b). The presence of this cryptic mutation raised the possibility that some of the phenotypes observed in the *PPO1*^*Δ*^*,2*^*Δ*^*,(3*^*1*^*)* flies could be due to the absence of PPO3. To clarify the function of the three PPOs, we separated *PPO3*^*1*^ from *PPO1*^*Δ*^ and *PPO2*^*Δ*^ by meiotic recombination. We also induced a null mutation in *PPO3* using CRISPR/Cas9, referred to as *PPO3*^*SK3*^, which is caused by a frameshift mutation in the first exon of the gene (Fig. 1a). Both the *PPO3*^*1*^ and *PPO3*^*SK3*^ mutations, which were generated in two distinct genetic backgrounds, show the same phenotype in all the experiments described below, indicating that *PPO3*^*1*^ is also a null mutation*.* As expected, *PPO3* mutants are perfectly viable and do not exhibit any overt developmental or pigmentation defect. Figure 1c shows that *PPO1*^*Δ*^*,2*^*Δ*^ and *PPO1*^*Δ*^*,2*^*Δ*^*,3*^*1*^ but not *PPO3*^*1*^ flies have a reduced life expectancy, confirming that the simultaneous presence of PPO1 and PPO2 is required for optimal fly longevity [2].

### PO1 and PO2 are the sole POs contributing to hemolymph injury-mediated melanization in larvae and adults

**Fig. 2:**
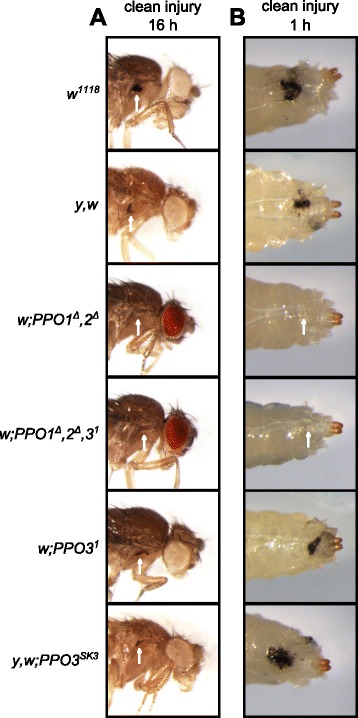
Both PPO1 and PPO2 but not PPO3 contribute to injury-related melanization in adults and larvae. Melanization of adults (**a**) and larvae (**b**) after clean injury is abolished only in the simultaneous absence of *PPO1* and *PPO2*. A normal melanization spot was observed in the two *PPO3* mutants. *Arrows* indicate the pricking site. Adults and larvae were wounded with a tungsten needle and blackening of the wound was recorded 1 h later in larvae and 16 h later in adults. A representative picture is shown for each genotype

**Fig. 3:**
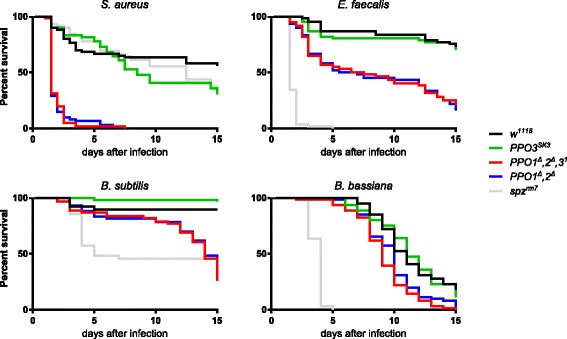
Contribution of PPO1,2 and PPO3 to host defense against bacteria and fungi. Survival rates of flies following septic injury with Gram-positive lysine-type bacteria (*Staphylococcus aureus* and *Enterococcus faecalis*), Gram-positive DAP-type bacterium (*Bacillus subtilis*) and natural infection with the entomopathogenic fungus *Beauveria bassiana*. Flies lacking the Toll ligand *spätzle* were used as immune-deficient controls. The *x*-axis is the time post-infection in days and the *y*-axis is the percentage of living flies. *PPO1* ^*Δ*^ *,2* ^*Δ*^ and *PPO1* ^*Δ*^ *,2* ^*Δ*^ *,3* ^*1*^ flies are less resistant to infections with *S. aureus* (*P* < 0.0001), *Enterococcus faecalis* (*P* < 0.0001), *Bacillus subtilis* (*P* < 0.0001) and *Beauveria bassiana* (*P* < 0.0005) compared to wild-type flies. Differences between *PPO3* ^*SK3*^ and *w* ^*1118*^ do not reach statistical significance. Data were analyzed by a log-rank test

**Fig. 4:**
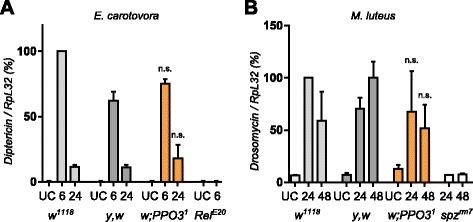
PPO3 is not required for Toll and Imd pathway activities. **a** Expression of *Diptericin* (*Dpt*) in *PPO* mutant flies. Total RNA was extracted from animals either uninfected or collected 6 h and 24 h after septic injury with Gram-negative bacteria *Erwinia carotovora*. Shown are the relative expression levels of *Dpt* in relation to *Rp49. PPO3* ^*1*^ flies show normal induction of *Dpt* expression levels. The Imd pathway mutant *Relish* was used as an immune-deficient control. **b** Expression of *Drosomycin* (*Drs*) in *PPO3* ^*1*^ mutant flies 24 h and 48 h after septic injury with the Gram-positive bacteria *Micrococcus luteus* shows that *PPO3* ^*1*^ mutant flies have a normal induction of *Drs*. The Toll pathway mutant *spätzle* ^*rm7*^ was used as an immune-deficient control. Shown are the relative expression levels of *Drs* in relation to *Rp49*. On the graphs, 100 % corresponds to the *Dpt* (*Drs*) expression level of wild-types flies 6 h (24 h) after septic injury*.* Data were analyzed using the Mann–Whitney test. Values represent the mean ± standard deviation of at least three independent experiments. *n.s.* not significant, *UC* unchallenged

Using a needle to injure wild-type larvae or adults induces a melanization spot at the wound site, the extent of which is usually proportional to the injury size. This blackening reaction results from de novo melanin synthesis catalyzed by PO and is further enhanced by the presence of microbial products [22]. Our previous study attributed all hemolymphatic PO activity to PPO1 and PPO2 due to the absence of melanization in the *PPO1*^*Δ*^*,2*^*Δ*^*,(3*^*1*^*)* stocks, while single *PPO1* or *PPO2* mutants showed only reduced or almost normal melanization, respectively. Consistent with this finding, no melanization spot on the cuticle of injured *PPO1*^*Δ*^*,2*^*Δ*^ and *PPO1*^*Δ*^*,2*^*Δ*^*,3*^*1*^ mutants was observed (Fig. 2a, b). In contrast, *PPO3* mutants show a wild-type level of melanization in injured larvae and adults. We next measured PO activity via the L-DOPA assay in hemolymph samples extracted from larvae. We found no significant difference in PO activity between *PPO3*^*1*^ and wild-type hemolymph samples. In contrast, hemolymph samples of *PPO1,2* deficient larvae show no PO activity, indicating that PPO3 alone is not sufficient to produce PO activity in hemolymph (Additional file 1: Figure S1). This confirms that PPO1 and PPO2 together produce all injury-mediated melanization in larvae and adults [2]. Survival analyses had shown that PO is required for resistance to microbial infection, notably to Gram-positive bacteria and fungi [2]. Survival analyses using the new fly lines now show that the strict *PPO1*^*Δ*^*,2*^*Δ*^ double mutation recapitulates all the phenotypes previously described using *PPO1*^*Δ*^*,2*^*Δ*^*,(3*^*1*^*)*: *PPO1*^*Δ*^*,2*^*Δ*^ flies are more susceptible to septic injury with the Gram-positive bacteria *Staphylococcus aureus*, *Enterococcus faecalis* and *Bacillus subtilis* and exhibit a mild susceptibility to natural infection with the fungus *Beauveria bassiana* (Fig. 3). In contrast, *PPO3*^*SK3*^ flies exhibit a wild-type level of resistance upon challenge with the same microbes. These experiments confirm the important role of melanization in fighting infection by Gram-positive bacteria and fungi, and are consistent with our previous results indicating that PPO1 and 2 are the sole sources for hemolymphatic PO. Additional data (Fig. 4a, b) show that *PPO3*^*1*^ does not markedly affect the Toll and Imd pathways in adults as revealed by the wild-type inducibility of *Diptericin* and *Drosomycin*, their respective read-out genes, in this mutant.

### *PPO3* is specifically expressed in lamellocytes

**Fig. 5:**
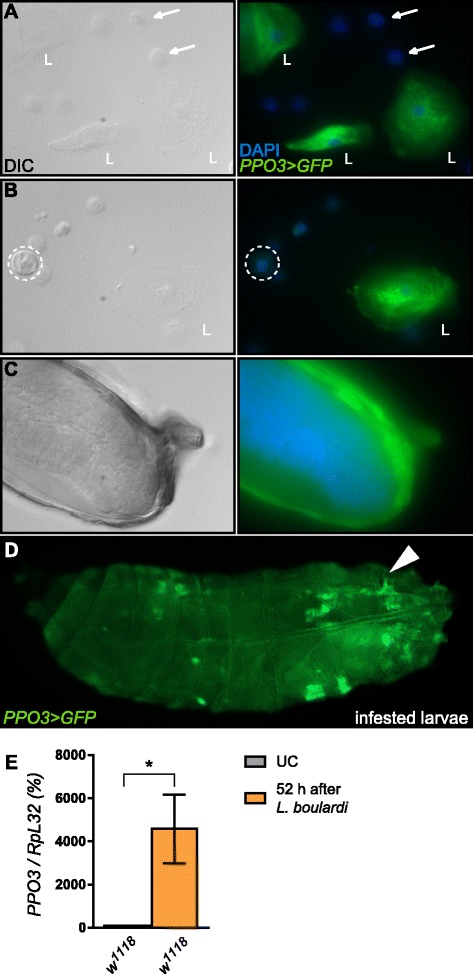
PPO3 is specifically expressed in lamellocytes. Differential interference contrast (*DIC*) and GFP fluorescence micrographs of hemocytes from larvae infested with *Leptopilina boulardi* expressing a *PPO3-Gal4*,*UAS-GFP* construct show that PPO3 is specifically expressed in all lamellocytes (*L*) either in circulation (**a**, **b**) or in the capsule surrounding the wasp egg (**c**). Plasmatocytes (*arrows*) and crystal cells (*dashed line*) do not express the reporter gene. GFP expression is *green* and DAPI staining is shown in *blue*. An overview of the whole infested larvae is shown in (**d**). The *arrow* indicates the site of the wasp larvae. Note that lamellocytes are found both around the egg and in circulation. **e** Quantitative reverse-transcription PCR (qRT-PCR) shows that *PPO3* gene expression is higher in larvae infested with *L. boulardi* compared to unchallenged larvae. In the graph, 100 % corresponds to *PPO3* expression levels of naive larvae. *DAPI* 4′,6-diamidino-2-phenylindole, *GFP* green fluorescent protein, *UC* unchallenged

*Drosophila* larvae have two types of hemocytes in the unchallenged state: plasmatocytes, which are macrophage-like cells, and crystal cells, which produce PPO1 and contain crystals of PPO2 [2, 19, 23, 24]. A third ‘inducible’ cell type, the lamellocytes, is produced only upon wasp infection or injury. Lamellocytes are large flat cells that differentiate in the lymph gland or from circulating plasmatocytes and adhere to foreign objects or aberrant tissue in a process called encapsulation. Capsules are usually melanized in a secondary step. The expression pattern of *PPO3* has been a source of conflicting observations. Irving et al. [17] reported its localization in lamellocytes using a microarray approach. In contrast, several studies [15, 16] report expression of *PPO3* in crystal cells. The observation that ubiquitous silencing of *PPO3* by in vivo RNAi suppresses melanization induced by the melanotic tumor *hop*^*Tuml*^ mutation, led Nam et al. [18] to hypothesize that *PPO3* is indeed expressed in lamellocytes. To distinguish between these two possibilities, we generated a reporter gene in which the yeast transcription factor GAL4 is under the control of 1.6 kb of *PPO3* upstream sequences. This transgenic line was crossed with *uas-GFP* flies to reveal the expression profile of *PPO3* using green fluorescent protein (GFP). We found no expression of *PPO3-Gal4* in plasmatocytes or crystal cells from naive larvae (data not shown). We then analyzed the expression pattern of *PPO3-Gal4;uas-GFP* in larvae upon infestation with the parasitoid wasp *Leptopilina boulardi*, which induces a massive production of lamellocytes. Figure 5a, b shows that the PPO3 reporter was not expressed in plasmatocytes or crystal cells in parasitized larvae, but was strongly expressed in all lamellocytes. High numbers of GFP positive lamellocytes were observed in circulation or around the wasp egg (Fig. 5c, d). No other tissue or cell expressed the reporter and no expression was detected in embryos and adults, consistent with the absence of lamellocytes at these two stages.

Consistent with these observations and previous reports [25, 26], qRT-PCR analysis indicated that the level of *PPO3* transcripts is low in unchallenged wild-type larvae but markedly up-regulated in larvae collected 52 h after wasp infestation, when lamellocytes have become abundant (Fig. 5e). While PPO1 and PPO2 are produced by crystal cells, our data clearly demonstrate that PPO3 is specifically expressed in lamellocytes. Since lamellocytes are absent in adults, this observation also explains why PPO3 does not contribute to hemolymphatic PO activity upon injury during this stage*.*

### Both PPO2 and PPO3 contribute to capsule melanization

**Fig. 6:**
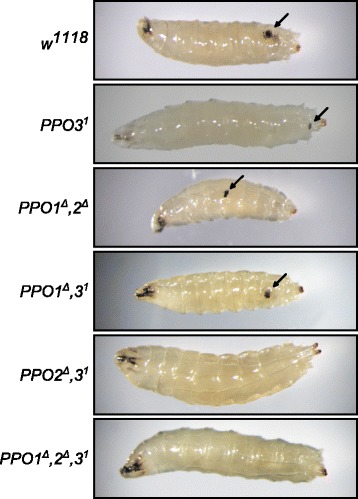
Both PPO3 and PPO2 contribute to melanization around a wasp capsule. Representative photos showing infested larvae containing eggs from a *Leptopilina boulardi* parasitoid wasp. Larvae mutant for *PPO3* ^*1*^ or *PPO1* ^*Δ*^ *,2* ^*Δ*^ show a melanized egg. No melanized capsule was found in *PPO2* ^*Δ*^ *,3* ^*1*^ and *PPO1* ^*Δ*^ *,2* ^*Δ*^ *,3* ^*1*^ infested mutant larvae. The presence of a non-melanized egg in *PPO2* ^*Δ*^ *,3* ^*1*^ and *PPO1* ^*Δ*^ *,2* ^*Δ*^ *,3* ^*1*^ mutant larvae was confirmed by subsequent dissection. *Arrows* indicate the position of the melanized wasp egg

A surprising result of Binggeli et al. was the observation that capsules around parasitoid wasp eggs were not melanized in *PPO1*^*Δ*^*,2*^*Δ*^*,(3*^*1*^*)* larvae [2]. This led us to conclude prematurely that PPO3 was not essential for the melanization process during encapsulation. The discovery of the cryptic *PPO3*^*1*^ mutation in the original stock and the specific expression of *PPO3* in lamellocytes prompted us to analyze further the role of PPO3 in the encapsulation process. Wild-type, *PPO3*^*1*^, *PPO1*^*Δ*^*,2*^*Δ*^ and *PPO1*^*Δ*^*,2*^*Δ*^*,3*^*1*^ second-instar larvae were infested by *Leptopilina boulardi*, and the presence of a melanized wasp egg was subsequently analyzed. Figure 6 shows the presence of melanized *L. boulardi* eggs in wild-type, *PPO1*^*Δ*^*,2*^*Δ*^ double-mutant and *PPO3*^*1*^ single-mutant third-instar larvae. In contrast, no melanized capsules were ever observed in the infested triple *PPO1*^*Δ*^*,2*^*Δ*^*,3*^*1*^ mutants (the presence of intact wasp eggs was checked by dissecting the larvae). Contradicting our previous conclusion, this result indicates that PPO3 does play a role in encapsulation that can be masked by the presence of PPO1 and/or PPO2. We then generated *PPO1*^*Δ*^*,3*^*1*^ and *PPO2*^*Δ*^*,3*^*1*^ double mutants to determine which of PPO1 or PPO2 contributes to capsule melanization together with PPO3. The result was unambiguous: we never found melanized capsules in *L. boulardi* infested *PPO2*^*Δ*^*,3*^*1*^ larvae while *PPO1*^*Δ*^*,3*^*1*^ larvae were phenotypically indistinguishable from *PPO3*^1^ (Fig. 6). Thus, two PPOs from different origins, PPO2 from crystal cells and PPO3 from lamellocytes, contribute to melanization that accompanies encapsulation.

**Fig. 7:**
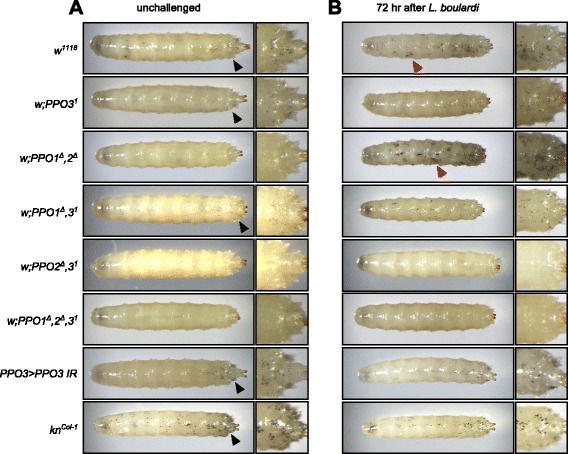
Phenoloxidase activity in PPO mutant larvae upon wasp infestation as revealed by heat treatment. Representative photos showing unchallenged larvae (**a**) and larvae infested by *Leptopilina boulardi* parasitoid wasps after heat treatment (**b**). Black dots on the cuticle correspond to melanized crystal cells due to PPO2 activity (*black arrows*, absent in *PPO2* mutants)*.* Large black patches correspond to melanized lamellocytes due to PPO3 activity (*red arrows*, absent in *PPO3* mutants). *Knot* ^*Col-1*^ larvae have no lamellocytes [28]

Heating larvae at 65 °C for 10 min induces the spontaneous activation of PPO [9]. As a consequence of this treatment, the population of sessile crystal cells that are attached underneath the integument can easily be visualized through the cuticle as black dots [27]. We used this method to investigate the respective contribution of the three PPOs to the melanization reaction. Consistent with the notion that black dots after heating are caused by the presence of PPO2 in crystal cells, *PPO1*^*Δ*^ and *PPO3*^*1*^ mutant larvae had melanized dots corresponding to the sessile crystal cells, but *PPO2*^*Δ*^ mutants did not (Fig. 7a). We then subjected third-instar larvae previously infested by *L. boulardi* to the heat treatment. In addition to the black dots corresponding to the spontaneous activation of PPO2 in crystal cells, wild-type larvae infested with *L. boulardi* show large black spots that were not observed in unchallenged larvae (Fig. 7b). The same treatment (infestation followed by heating) applied to the various PPO mutants suggests that the large black spots are due to PPO3 activity in lamellocytes, as they are totally absent in *PPO3*^*1*^ larvae. Our conclusions were further strengthened by the observation that silencing *PPO3* by RNAi in lamellocytes alone using the *PPO3-Gal4* driver phenocopied the *PPO3*^*1*^ phenotype. Furthermore, a *knot* mutant (also called *collier*), which cannot produce lamellocytes [28], shows a melanization pattern similar to the *PPO3*^*1*^ mutants following wasp infestation.

### Melanization linked to encapsulation is not dependent on Hayan or Sp7

It has been proposed that PPO3 is produced in its active form, while PPO1 and PPO2 are synthesized as zymogens, which are cleaved by SP activity to generate the active form [18, 20]. This cleavage is thought to be mediated by a clip-domain SP named Hayan [22]. Hayan also exists as a zymogen that is itself stimulated through a stepwise process involving other SPs. One upstream clip-domain SP is SP7 (also called MP2), which is specifically expressed in crystal cells [12–14, 29]. Indeed, while a null mutation in *Hayan* totally abolishes hemolymphatic PO activity in adults [22], a partial *Sp7* loss-of-function, *Sp7*^*PAE1*^, slightly reduces it [14]. To date, the roles of Hayan and Sp7 in the encapsulation reaction in larvae have not been fully clarified.

**Fig. 8:**
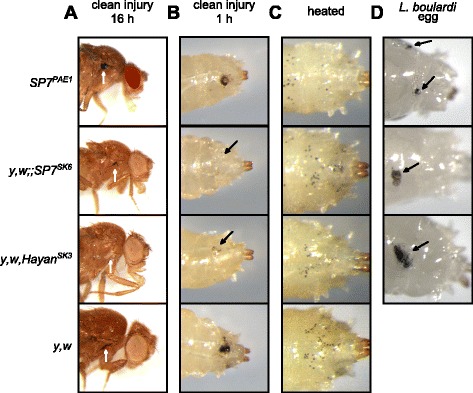
Hayan and SP7 do not contribute to melanization of a wasp capsule. Melanization of adults (**a**) after clean injury is abolished only in the *Hayan* ^*SK3*^ mutant. A slightly reduced melanization spot was observed in the *Sp7* ^*SK6*^ mutant. In contrast, after clean injury of larvae (**b**), melanization is abolished only in *Sp7* ^*SK6*^ whereas *Hayan* ^*SK3*^ shows residual melanization. *Arrows* indicate the pricking site. Adults and larvae were wounded with a tungsten needle and blackening of the wound was recorded 1 h later in larvae and 16 h later in adults. After heat-treating larvae (**c**), black dots, corresponding to crystal cells, appeared in *Hayan* ^*SK3*^ and *Sp7* ^*SK6*^ comparable to wild-type controls. Infested larvae containing eggs of *Leptopilina boulardi* parasitoid wasps (**d**) show the presence of a melanized egg in *Hayan* ^*SK3*^ and *Sp7* ^*SK6*^. *Arrows* indicate the position of the melanized wasp egg. A representative picture is shown for each genotype

To address the role of SP cleavage in larval and adult melanization, we first generated using CRIPR/Cas9 two null mutations in *Hayan* and *Sp7* and analyzed their contribution to the melanization reaction using the same techniques as described above. We confirmed that both *Sp7*^*SK6*^ and *Hayan*^*SK3*^ mutations affect melanization at an injury site, with slight differences according to the stage of the animal. As expected, the newly generated null *Sp7* mutant, *Sp7*^*SK6*^, induces a much stronger phenotype than the previously described hypomorphic *SP7*^*PAE1*^ allele: *Sp7*^*SK6*^ mutant adults show a very weak melanized spot at the injury site compared to *SP7*^*PAE1*^ (Fig. 8a). As expected [22], the *Hayan*^*SK3*^ mutation totally abolishes melanization at the injury site in adult flies. Interestingly, injured *Hayan*^*SK3*^ and *Sp7*^*SK6*^ larvae had opposite phenotypes compared to mutant adults. Whereas *Sp7*^*Δ*^ larvae do not develop any melanization at the injury site, *Hayan*^*SK3*^ larvae still display a very weak blackening reaction (Fig. 8b). However, both *Sp7*^*SK6*^ and *Hayan*^*SK3*^ larvae showed a wild-type pattern of melanized black dots upon heating at 65 °C (Fig. 8c). This result indicates that heat treatment can induce spontaneous activation of PPO in crystal cells in the absence of upstream SPs. Finally, we observed that both *Sp7*^*SK6*^ and *Hayan*^*SK3*^ mutant larvae can produce melanized capsules around *L. boulardi* eggs (Fig. 8d). This indicates that Hayan and Sp7 are not mandatory for the melanization around the capsule, although a minor contribution of these SPs cannot be excluded. In summary, the loss of *Hayan* or *Sp7* both mimic the *PPO1*^*Δ*^*,2*^*Δ*^ phenotype, pointing to a major role of these SPs following septic injury. The formation of wild-type melanized capsules in both SP mutants is consistent with the observation that PPO3 does not need cleavage to become active.

### PPO3 arose by gene duplication from PPO2 during radiation of the *D. melanogaster* group

To place the functional differences among *Drosophila PPO* genes in an evolutionary context, we reconstructed the phylogeny of the PPO gene family using genome sequences from 23 species of *Drosophila* [30, 31]. We confirmed previous observations by Salazar-Jaramillo et al. [26] that the *PPO1* and *PPO2* genes are conserved across all *Drosophila* genomes sequenced, whereas *PPO3* is restricted to the *D. melanogaster* group (Additional file 2: Figure S2). By analyzing a larger panel of species in the *D. melanogaster* group than in previous work [26], we found that a canonical *D. melanogaster*-like *PPO3* gene can only be found in the *D. melanogaster* subgroup (containing *D. melanogaster*, *D. simulans*, *D. sechellia*, *D. erecta* and *D. yakuba*) and *D. eugracilis*, but not in other members of the *D. melanogaster* group or species outside this group. Evidence for a partial *PPO3* gene can be found in *D. takahashii* (Additional file 2: Figure S2), which may indicate that a *PPO3*-like gene was present in the ancestor of these species. All *Drosophila* species with canonical (or partial) *PPO3* genes are able to encapsulate parasitoid wasps melanotically and to produce lamellocytes [26, 32, 33]. However, additional species in the *D. melanogaster* group (i.e., *D. ficusphila*) are also able to cellularly encapsulate wasps and produce lamellocytes [34], suggesting that the acquisition or retention of *PPO3*-based melanization in lamellocytes occurred after the cellular basis for encapsulating parasitoid wasps evolved.

Phylogenetic evidence for all *PPO* genes in *Drosophila* shows the *PPO3* clade clusters more closely with *PPO2* than with *PPO1* (Additional file 3: Figure S3; see also [8]), consistent with the functional overlap between *PPO2* and *PPO3* reported here. Moreover, both *PPO3* and *PPO2* have a four-exon gene structure, whereas *PPO1* has six exons. The *PPO* gene tree also shows that all *PPO3* genes cluster together outside the *PPO2* clade, which is consistent with two hypotheses for the timing of the *PPO3* duplication event: (i) the *PPO3* gene arose prior to the diversification of the *Drosophila* and *Sophophora* subgenera and was subsequently lost in multiple lineages, or (ii) the *PPO3* gene arose in the *D. melanogaster* group and underwent a transient period of accelerated sequence evolution shortly after duplication. Assuming the species tree in Ometto et al. [35], an ancient origin is unlikely because it requires one gain and at least seven independent losses in different *Drosophila* lineages, while simultaneously invoking selective maintenance of an ancestral *PPO3* prior to the evolution of the lamellocyte cell type on the lineage leading to the *D. melanogaster* group. A recent origin is more parsimonious and only requires a single evolutionary event followed by an accelerated rate of evolution in one of the genes after duplication (in this case *PPO3*), a pattern that has been observed for duplicate genes in yeast and mammals [36]. Thus, we conclude a *PPO2*-like ancestral gene was likely the source for the *PPO3* gene duplication event, and that this event most likely occurred recently during the radiation of the *D. melanogaster* group species.

The maximal genomic extent of the *PPO3* duplication (inferred by alignment with *D. ananassae*, the closest outgroup species that lacks this locus and has a high-quality genome assembly) does not fully contain the sequence in our *PPO3-Gal4* reporter construct that is needed to give lamellocyte expression (Additional file 2: Figure S2). In fact, the region of our reporter that extends the regulatory region reported by Ferjoux et al. [16] and likely contains lamellocyte-specific regulatory elements lies within the neighboring gene CG9890, which is conserved in *D. ananassae*. Thus, it is likely that regulatory sequences responsible for lamellocyte expression were not a part of the ancestral locus that gave rise to *PPO3*, but rather arose during or after the *PPO3* duplication event.

After the gain of *PPO3*, *D. sechellia* subsequently lost the ability to produce lamellocytes and melanotically encapsulate parasitoids [26, 32, 33]. Salazar-Jaramillo et al. [26] found an accelerated rate of evolution in the *D. sechellia PPO3* gene (Dsec\GM15980), which they proposed reflected relaxation of selective constraints on *PPO3* associated with the loss of the melanotic encapsulation phenotype. We found no evidence for an increased rate of evolution of the *D. sechellia PPO3* gene in our data, and tracked this discrepancy to a gene model error in FlyBase that fuses incomplete versions of *PPO3* and a neighboring gene (CG44252) in *D. sechellia* that was used by Salazar-Jaramillo et al. [26]. We did, however, find evidence for an inactivating mutation in the *D. sechellia PPO3* gene at amino acid position 48, which converts the terminal glutamine residue of the propeptide region to a stop codon, and is predicted to generate a truncated version of the *PPO3* protein. This loss-of-function coding-sequence mutation together with loss of *PPO3* expression in *D. sechellia* [26] supports the general model that PPO3 functions specifically in lamellocytes, and that the proper cellular context for its function in melanotic encapsulation is required to maintain selective constraint on this locus.

## Discussion

Gene duplication is recognized as an important process in evolution. About 40 % of the 13,601 *Drosophila* genes are duplicates of other genes [37]. Duplication is often associated with sub-functionalization in which each of the daughter genes adopts part of the function of the parental gene or neo-functionalization in which duplication is at the origin of a novel function [37]. Gene duplication can lead to the formation of a large gene family. Both animals and plants harbor large families of genes devoted to immune defense. Immune genes encoding effector or recognition molecules often exist in multiple copies, while genes encoding signaling pathway components are typically single copy [38, 39]. It is expected that a large repertoire of immune molecules confer broader recognition and effector capacity as well as more regulatory flexibility in the use of this repertoire. Thus, the higher number of genes encoding peptidoglycan recognition proteins or antimicrobial peptides in the fruit fly *Drosophila* compared to the pea aphid can easily be understood by their respective environments: aphids feed on sap, a rather sterile diet, while *Drosophila* feed on rotting fruits swarming with microbes [40]. Besides these general statements, the *raison d’être* of the size of an immune gene family remains speculative.

Phenoloxidases are rate-limiting enzymes, which determine the extent of melanization, and as such can be considered as effector immune molecules [8]. The number of *PPO* genes is variable among insects, ranging from one in the bee *Apis mellifera* to ten in the mosquito *Aedes aegypti* [8]. In this work and in Binggeli et al. [2], we have generated loss-of-function mutations in the three *PPO* genes of *Drosophila* and analyzed the contribution of each of them to immune reactions. Our gene deletion and double-mutant analysis allowed us to ascribe specific functions to each of the three PPOs. Our study confirms that two crystal cell PPOs, PPO1 and PPO2, contribute to the bulk of melanization induced by injury, with PPO1 immediately available and PPO2 being deployed later [2]. Melanization is one of the most rapid immune responses, as the dark spot is visible as soon as 10 min after injury. This suggests a key role for this mechanism in the early steps of wound healing and pathogen control. This could explain why PPOs are rather stored as ready-to-use proteins and not regulated at the transcriptional level. The existence of crystal cells, whose sole reported function so far is melanization, provides *Drosophila* with an efficient way to store and quickly release this key enzyme. Recent studies have shown that crystal cells either derive from progenitor blood cells or can differentiate from plasmatocytes [15, 27, 41, 42]. Thus, *Drosophila* can indirectly modulate the amount of PPO1 and PPO2 by regulating crystal cell differentiation*.* The observation that the *PPO1* deletion does not affect crystals in crystal cells led us to propose that PPO2 is the main source of crystalline PPO and that PPO1 is either localized in the cytoplasm of crystal cells or continuously secreted into the hemolymph [2]. The extreme fragility of crystal cells did not allow us to clarify this point.

Our work here demonstrates that PPO3 is restricted to lamellocytes and contributes to the encapsulation of a wasp egg. This explains why the *PPO3* transcripts are present at barely detectable levels in unchallenged larvae, which contain few or no lamellocytes. An intriguing observation is that two PPOs, PPO2 and PPO3, contribute to the melanization of the capsule formed around a wasp egg. This means that it is achieved by phenoloxidases derived from two hemocyte types: crystal cells and lamellocytes. Lamellocytes are large flat cells with adhesive properties that can bind to wasp eggs and form multilayers. Our study suggests that crystal cells could also be guided to the capsule where they would release PPO2 crystals close to the encapsulated egg. It is still unknown whether crystal cells have the ability to stick to non-self elements or if they need the assistance of other hemocytes to reach their target as suggested by [27]. The absence of any marked role of PPO1 in encapsulation is puzzling*.* We speculate that localization of PPO1 in the hemolymph, which remains to be assessed, could explain why PPO1 is not involved in encapsulation. PPO in circulation would not have the ability to be directed to a foreign element such as a wasp egg, but could be immediately activated by the presence of oxygen at a wound site.

**Fig. 9:**
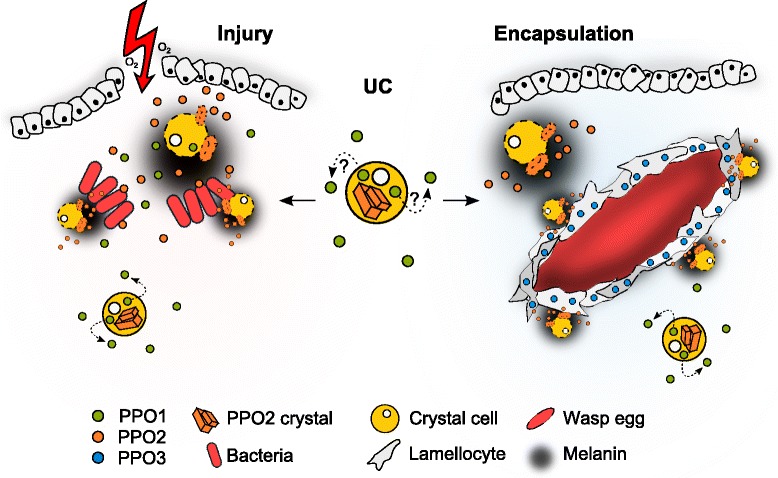
Model of phenoloxidase activation after injury and wasp infestation. Both PPO1 and 2 are synthetized by crystal cells. In the unchallenged condition, PPO2 is stored in the crystal cells while the localization of PPO1 is still unknown (here shown in the hemolymph) (*middle*). After a clean or septic injury, PPO1 and PPO2 are the only source of hemolymphatic PO activity (*left*). After parasitic infestation, PPO3 is produced by lamellocytes and contributes with PPO2 to the melanization around the wasp egg during encapsulation (*right*). *UC* unchallenged

Combining the various mutations allowed us to show that, while each single *PPO* mutant has a specific phenotype, knocking out two genes out of three is required to abolish fully a specific function. Thus, *Drosophila* PPOs have partially overlapping functions to optimize melanization in at least two stress conditions, injury and parasitization (Fig. 9). Future studies should investigate the role of these PPOs in the melanization of organs such as the gut, salivary gland and trachea, which is sometimes observed upon oral infection or in a tumorous-like state [43, 44]*.* The selective pressure exerted by parasitoid wasps on *D. melanogaster* has probably led to the development of dedicated cellular immune defenses, with the emergence of inducible adhesive cells with PPO3 (lamellocytes), storage cells (crystals cells with PPO2) and hematopoietic organs (the lymph gland and sessile islets). All these elements are activated upon parasitization and contribute collectively to capsule formation by mechanisms that are still poorly understood [45].

Most *Drosophila* species possess two *PPO* genes, *PPO1* and *PPO2*, except for some species from the *D. melanogaster* group (*D. melanogaster*, *D. simulans*, *D. sechellia*, *D. erecta*, *D. yakuba* and *D. eugracilis*), which have three (Additional file 2: Figure S2). Since the *PPO3* gene sequence, structure and function are more similar to those of *PPO2* than of *PPO1*, we propose that a duplication of an ancestral *PPO2*-like gene gave rise to an ancestral *PPO3*-like gene in the common ancestor of the lineage leading to *D. eugracilis* and the *D. melanogaster* subgroup. This was followed by a functional diversification with *PPO2* maintaining expression in crystal cells and *PPO3* becoming expressed only in lamellocytes. This scenario is consistent with a period of accelerated evolution of the *PPO3* lineage after duplication (Additional file 3: Figure S3). Future studies in other *Drosophila* species might shed light on whether this gene duplication event led to sub-functionalization (the ancestral gene was expressed in both crystal cell and lamellocyte lineages) or neo-functionalization (the ancestral gene was only expressed in crystal cells and the duplicate gene acquired a new expression pattern in lamellocytes). The observation that the existence of a third PPO is restricted to the *D. melanogaster* group suggests that massive production of PO emerged as a crucial mechanism to reinforce the defense against parasitoids in this lineage. The same selective pressure has probably led to other anti-parasite defenses. Recent studies have shown that wasp encapsulation in *D. ananassae* (a species outside the *D. melanogaster* subgroup) is mediated by another mechanism involving multinucleated giant hemocytes, that are formed by the aggregation of hemocytes [46]. The capsule around the wasp is not melanized in this species [26, 33, 46]*.* The melanization of the capsule points to a critical role of melanization against parasites. This is also supported by studies showing that virulent wasps have suppressive mechanism against melanization, such as the injection of serpins [47]. We performed survival analysis with *PPO* mutants using a highly virulent wasp species, *L. boulardi*, and a weakly virulent one, *L. clavipes*. The results did not reveal any striking effect although there was a trend for a lower resistance of larvae to *L. clavipes* infestation in the absence of PPO (Additional file 4: Figure S4). Consistent with this, we noticed that it is easier to cultivate the wasp *Asobara tabida* using *PPO1,2,3* flies compared to wild type. Taking possible effects of the genetic backgrounds into consideration, the results of our survival analysis in the laboratory should be taken with caution. It is possible that the optimum level of melanization for successful encapsulation changes in an ongoing arms race requiring a very tight balance, as melanization is also toxic for flies. This would explain why *PPO3* mutants tend to be more susceptible than *PPO1,2,3* deficient flies upon infection with *L. clavipes* (Additional file 4: Figure S4). Thus, the relevance of PPO in wasp encapsulation requires further analysis that should take into account the influence of the genetic background and the use of a more natural setting of infection.

The mechanisms of activation of PPO in *Drosophila* are not yet fully understood. Previous studies suggested that PPO1 and PPO2 require cleavage by SPs while PPO3 would be constitutively active. Indeed over-expression of PPO3 is sufficient to induce melanization of tissues as diverse as the eye or the salivary gland [18]. Here, we show that mutations in *Hayan* or *SP7* strongly reduce melanization due to septic injury but do not affect melanization around a wasp capsule. This suggests that either PPO3 does not require proteolytic cleavage, as suggested by molecular modeling and in vitro testing of PPO3 mutants [20], or its maturation requires distinct SPs. Interestingly in this context, heat treatment of wasp-infected larvae produced melanized cell aggregates, which corresponds to PPO3 activity in lamellocytes. This indicates that PPO3 is present in lamellocytes in an inactive form that can be activated by heat. Thus, the possibilities are that PPO3 needs an additional step to be activated, it is produced together with an inhibitor, or it is inactive due to the absence of its substrate. The mechanism by which PPO3 is activated only in lamellocytes around a capsule remains to be deciphered.

This analysis started with the serendipitous discovery of a *PPO3* deletion in the *PPO1,PPO2* double-mutant fly stock. Since this deletion was also found in a *white* control stock, we assume that this mutation was introduced when backcrossing *PPO1*. Since fly stocks in the laboratory are cultivated in the absence of parasitoid pressure, it cannot be excluded that this gene quickly pseudogenizes as it has no role beyond encapsulation. We were fortunate that the presence of this mutation does not affect the main conclusion of Binggeli et al. [2], which states that PPO1 and PPO2 are responsible for all hemolymphatic PO activity. The absence of melanization around the wasp egg in *PPO1*^*Δ*^*,2*^*Δ*^*,(3*^*1*^*)* larvae led us to miss the important contribution of PPO3 to encapsulation. This underlines that one of the main issues in generating an extensive characterization of the *Drosophila* immune system is that of the genetic background and namely what we refer to as ‘wild type’. Fly geneticists have usually a number of ways to assess the effect of the genetic background by analyzing the phenotype caused by mutations in different contexts. This can be done by placing the mutation over a deficiency or by extensive backcrossing to generate isogenic stocks as recently described in [48]. An important task should be to analyze how the studied phenotype varies in diverse backgrounds. In this study, the phenotypes of each of the *PPO* single mutations were clear enough and in accordance with their expression patterns, so that we could be confident about their respective functions. The striking phenotypes of *PPO1*^*Δ*^*,2*^*Δ*^ and *PPO2*^*Δ*^*,3*^*1*^ double mutants, which fail to melanize upon injury and wasp infection, respectively, reinforced our conclusions, which would have been more difficult to draw from only single-mutant analysis. Thus, generation of multiple mutations in combination is an adequate approach to assess better the function of gene families, notably those involved in effector mechanisms. Similar studies on other immune gene families should provide insights into the organization of the *Drosophila* immune system and that of other organisms.

## Conclusions

We conclude that differences in spatial localization, immediate or late availability, and mode of activation underlie the functional diversification of the three *Drosophila* PPOs, with each of them having non-redundant but overlapping functions.

## Methods

### Insects stocks and mutant generation

Unless indicated otherwise, *w*^*1118*^ or *y*^*1*^*w*^*1118*^ flies were used as wild-type controls. The *PPO1*^*Δ*^, *PPO2*^*Δ*^, *PPO1*^*Δ*^*,2*^*Δ*^*,(PPO3*^*1*^*)*, *Relish*^*E20*^ (*Rel*^*E20*^), *spätzle*^*rm7*^ (*spz*^*rm7*^) and *SP7*^*PAE1*^ lines were described previously [2, 10, 14, 49]. *kn*^*col-1*^ lamellocyte deficient larvae were obtained by using the w*; kn^*col-1*^*;P{col5-cDNA.C}* fly line that carries the lethal mutation *knot* and a *knot* transgene with a restricted expression pattern rescuing this lethality [28]. w*; P{10XUAS-mCD8::GFP}attP2 (*UAS-GFP*) lines was obtained from the Bloomington Stock Center. The parasitoid wasp *L. boulardi* (kindly provided by M Crozatier) was reared on *PPO1*^*Δ*^*,2*^*Δ*^*,3*^*1*^ triple-mutant fly stocks at room temperature. After emergence, wasps were kept at room temperature and provided with honey until used for experiments. The *PPO3*^*SK3*^, *Hayan*^*SK3*^ and *Sp7*^*SK6*^, mutant lines were generated by CRISPR/Cas9 as described in [21] (Additional file 5: Figure S5). The *uas-PPO3-IR* (ch II) was obtained from VDRC (50737). The *PPO3-Gal4* line was generated by cloning a 1.6 kb sequence upstream of the *PPO3* gene (*PPO3-Gal4* forward 5′-TTGAGGGCGGTGAAGTTGTC-3′, reverse 5′- GGAGGACCTTTAGCGAGCAG-3′) into pBPGUw vector [50] followed by PhiC31 integrase-mediated transgenesis (BestGene Inc). The *PPO1*^*Δ*^*,2*^*Δ*^, *PPO1*^*Δ*^*,3*^*1*^ and *PPO2*^*Δ*^*,3*^*1*^ lines were generated by reintroducing the corresponding wild-type alleles into the *PPO1*^*Δ*^*,2*^*Δ*^*,3*^*1*^ mutant by meiotic recombination. *Drosophila* stocks were maintained at 25 °C on standard fly medium.

### Microorganism culture and infection experiments

The bacterial strains used and their respective optical density of the pellet (OD) at 595 nm were: the Gram-negative bacteria *Erwinia carotovora* (*Ecc15*, OD 200); the DAP-type peptidoglycan (PGN) containing Gram-positive bacteria *Bacillus subtilis* (OD 5); and the Lys-type PGN containing Gram-positive bacteria *Micrococcus luteus* (*M. luteus*, OD 200), S*taphylococcus aureus* (*S. aureus*, OD 0.5) and *Enterococcus faecalis* (OD 0.5). Strains were cultured in Luria broth at 29 °C (*Erwinia carotovora* and *M. luteus*) or 37 °C (other species). Spores of entomopathogenic strains *Beauveria bassiana 802* were grown on malt agar plates at 29 °C for approximately 3 weeks until sporulation [49]. Systemic infections (septic injury) were performed by pricking adult females in the thorax with a thin needle previously dipped into a concentrated pellet of bacteria. Natural infections were initiated by shaking anesthetized flies in a Petri dish containing a sporulating culture of entomopathogenic fungi *Beauveria bassiana*. Infected flies were subsequently maintained at 29 °C (*Erwinia carotovora*, *M. luteus* and *Beauveria bassiana*) or at 25 °C (all other bacteria). At least three tubes of 20 flies were used for each survival experiment and survival was scored daily. For lifespan experiments, flies were kept on normal fly medium and were flipped every 2 days.

### Wounding experiment

A ‘clean’ injury was an injury performed with a sterilized needle. A low level of bacterial contamination is still possible since the surface of the insect was not sterilized. For imaging of the melanization reaction upon pricking, the thorax of the animal was pricked (as described in infection experiments) using a sterile needle (diameter ~5 μm). Pictures were taken 16 h post-pricking. Third-instar larvae were pricked dorsally near the posterior end, using a sterile needle (diameter ~5 μm). Pictures of melanized larvae were taken 1 h post-injury. Pictures were captured with a Leica DFC300FX camera and Leica Application Suite. For publication purposes, brightness and contrast were increased on some images.

### Live imaging and immunofluorescence

For hemocyte imaging, larvae were cleaned in phosphate-buffered saline (PBS) and dissected on silane-coated microscope slides (Tekdon, Inc) into a drop of 1 % bovine serum albumin-PBS. Hemocytes were allowed to settle for 30 min, then fixed with 4 % paraformaldehyde-PBS for 15 min and rinsed with PBS.

For staining of capsules from wasp-infested larvae, capsules were dissected 3 days after infection, fixed with 4 % paraformaldehyde-PBS and rinsed with PBS. Samples were observed for fluorescence with an Axioplot imager Z1 and Axiocam mRM camera (Zeiss).

### PO activity

Hemolymph was collected by dissecting larvae in 4 °C PBS. The protein concentration was adjusted after a Bradford test. Sample volumes were adjusted in 20 μl of 5 mM CaCl_2_ solution. After addition of 80 μl L-DOPA solution (20 mM, pH 6.6), the samples were incubated at 29 °C in the dark. The OD at 492 nm was then measured. An L-DOPA solution without hemolymph was used as a blank. Each experiment was repeated three times.

### Quantitative reverse-transcription PCR

For quantification of mRNA, whole flies or larvae were collected at indicated time points. Total RNA was isolated from 10–15 adult flies or larvae using TRIzol reagent and dissolved in RNase-free water. Next, 1 μg of total RNA was reverse-transcribed in 10 μl reaction volume using PrimeScript RT (TAKARA) with random hexamer and oligo dT primers. Quantitative PCR was performed on a LightCycler 480 (Roche) in 96-well plates using the LightCycler 480 SYBR Green I master mix or on a LightCycler 2.0 (Roche) in capillaries using dsDNA dye SYBR Green I (Roche). Primers were as follows: *Diptericin* forward 5′- GCTGCGCAATCGCTTCTACT-3′, reverse 5′-TGGTGGAGTGGGCTTCATG-3′; *Drosomycin* forward 5′-CGTGAGAACCTTTTCCAATATGAT-3′, reverse 5′- TCCCAGGACCACCAGCAT-3′; *Rp49* forward 5′-GACGCTTCAAGGGACAGTATCTG-3′, reverse 5′-AAACGCGGTTCTGCATGAG-3′; and *PPO3* forward 5′-GGCGAGCTGTTCTACT-3′, reverse 5′- GAGGATACGCCCTACTG-3′.

### Wasp infestation and quantification of fly survival to wasp infestation

For wasp infections, 30 synchronized second-instar wild-type or mutant larvae were placed on a pea-sized mound of fly food within a custom-built wasp trap in the presence of three female wasps for 2 h (*L. boulardi*) or 72 h (*L. clavipes*). For survival experiments, parasitized larvae were kept at room temperature and scored daily for flies and wasps. The difference between enclosed flies and wasps to the initial number of larvae was set as dead larvae/pupae. For imaging of lamellocytes or wasp eggs, infested larvae were dissected 72 h after being exposed to wasps. For qRT-PCR of PPO3, the total RNA of larvae was isolated 52 h after wasp infection.

### Heating of larvae to induce spontaneous melanization

Larvae were heated in PBS at 67 °C for 20 min to induce the spontaneous activation of PPO within hemocytes [9, 27].

### PPO sequence analysis

Predicted orthologs of *PPO* genes from 23 species in the *Drosophila* genus were identified by extracting and concatenating exonic regions from the UCSC Genome Bioinformatics Database multiz27way insect alignments [51]. Genomic alignments extracted from UCSC for each gene were cross-referenced with orthology predictions for 12 *Drosophila* genomes from OrthologDB [52] and in three cases where UCSC genomic alignments lacked a gene model for a particular species (*PPO1* from *D. mojavensis*, *PPO1* from *D. grimshawi* and *PPO2* from *D. ananassae*), the FlyBase gene model indicated by OrthologDB was added to the alignment [30, 53].

Sequences for all three *PPO* genes were combined and aligned using Clustal Omega [54] using default settings in SeaView 4.0 [55]. A preliminary phylogenetic tree was produced using the BioNJ algorithm [56] in SeaView. *PPO* sequences clustering in the incorrect gene or species clade were inspected in the UCSC Genome Browser. Three predicted *PPO* genes based on the UCSC alignments (*PPO1* for *D. miranda*, *PPO1* for *D. kikkawai* and *PPO3* for *D. kikkawai*) clustered incorrectly in the *PPO2* clade. Inspection of global alignment nets and chains in the UCSC browser revealed these to be alignment artifacts. Likewise, systematic inspection of nets and chains for all *D. melanogaster* group species revealed that a predicted *PPO3* sequence from *D. takahashii* based on UCSC whole-genome alignment was composed of two non-syntenic fragments, and there was no evidence for a *PPO3* sequence in the two species most closely related to *D. takahashii* (*D. biarmipes* and *D. suzukii*). Thus, these four sequences were removed from the final alignment before producing the final phylogenetic tree using RAxML version 8.0.26 [57]. Maximum-likelihood tree searches were conducted using a general time-reversible model of nucleotide substitution with Γ rate heterogeneity, with all model parameters estimated by RAxML. Trees were inferred using a combined approach, with an initial 100 bootstrap replicates and a full maximum-likelihood search for the best-scoring tree, using the rapid bootstrap algorithm [58]. The best-scoring maximum-likelihood tree was visualized and annotated in FigTree version 1.4.2 [59].

The PPO3 gene overview was adapted with FancyGene [60]. The protein structure of PPO3 wild type and PPO3^1^ was predicted by Phyre2 [61] and visualized using the VMD suite [62]. All structural illustrations were rendered in VMD.

### Statistical analysis

Each experiment was repeated independently a minimum of three times (unless otherwise indicated). Error bars represent the standard deviation of replicate experiments (unless otherwise indicated). Statistical significance of survival data was calculated with a log-rank test, and *P* values are indicated in figure legends. Statistical significance of PO activity was calculated with two-way ANOVA with Tukey correction. Otherwise, statistical significance was calculated using the Mann–Whitney test and *P* values of <0.05 (indicated by *), <0.005 (indicated by **) and <0.0005 (indicated by ***) were considered significant.

## Additional files

Additional file 1: Figure S1. PPO3 does not contribute to hemolymph PO activity. Hemolymph PO activity of unchallenged larvae was monitored with an L-DOPA assay. Spontaneous PO activity is not affected in *PPO3* ^*1*^ mutant larvae compared to wild-type larvae. The hemolymph of wandering larvae was collected and examined for PO activity by transferring L-DOPA to dopachrome. The hemolymph of *PPO1,2,3* mutant larvae was used as a negative control. Differences between *PPO3* ^*1*^ and the *w* ^*1118*^ wild-type control do not reach statistical significance. Data were analyzed by two-way ANOVA with Tukey correction. Values represent the mean ± standard error of the mean of three independent experiments. *OD* optical density (PDF 20 kb) Additional file 2: Figure S2. Comparative genomics of the *PPO3* locus. Shown are the best pairwise genome alignments for 22 *Drosophila* species versus *D. melanogaster* based on the UCSC genome browser net tracks. Species in the *D. melanogaster* group plus *D. eugracilis* have only one net that aligns contiguously the entire *PPO3* microsyntenic region. Conversely, other species (besides *D. takahashii*) have a gap in the alignment at *PPO3* that is filled by a secondary net that arises from alignment to paralogous sequences elsewhere in the genome. *D. takahashii* has only a single alignment net in the *PPO3* region; however, this net is fragmented precisely in the *PPO3* gene, consistent with the possible presence of non-canonical *PPO3*-like gene sequences in this species (PDF 1061 kb) Additional file 3: Figure S3. Phylogenetic tree of PPO genes from *Drosophila* species. A maximum likelihood phylogenetic tree was constructed with the exonic sequences of *PPO1*, *PPO2* and *PPO3* using RAxML. Nodes with more than 90 % bootstrap support are labeled. Branch lengths are in substitutions per site. The tree is midpoint rooted and based on a multiple alignment of all *PPO* genes from *D. melanogaster*, *D. simulans*, *D. sechellia*, *D. yakuba*, *D. erecta*, *D. eugracilis*, *D. suzukii*, *D. biarmipes*, *D. takahashii*, *D. rhopaloa*, *D. elegans*, *D. ficusphila*, *D. kikkawai*, *D. bipectinata*, *D. ananassae*, *D. pseudoobscura*, *D. persimilis*, *D. miranda*, *D. willistoni*, *D. mojavensis*, *D. virilis*, *D. albomicans* and *D. grimshawi*. The placement of the root in our tree is supported by a previous phylogenetic analysis of *PPO* genes across arthropods [8] (PDF 182 kb) Additional file 4: Figure S4. Survival analysis after *Leptopilina clavipes* and *L. boulardi* wasp infestation in wild type and *PPO* mutants. Synchronized L2 larvae were exposed to gravid female *L. clavipes* (A) or *L. boulardi* (B) wasps in custom infection traps, re-isolated and cultured at room temperature until wasps emerged. Vials were scored daily for total number numbers of eclosed flies and wasps. *PPO3* mutant larvae show enhanced susceptibility against *L. clavipes*, whereas the loss of all PPO does not further increase this effect. The mild effect of PPO mutants on wasp encapsulation led us to hypothesize that optimal encapsulation requires a precise level of phenoloxidase activity due to a possible toxic effect on the host (PDF 41 kb) Additional file 5: Figure S5. Schematic representation of novel CRISP/Cas9 mutants. Overview of the Hayan (A) and *Sp7* (B) genes. *Arrows* indicate the position of the mutations. Partial sequences are shown, and deleted nucleotides of *Hayan* ^*SK3*^ and *Sp7* ^*SK6*^ are marked in *red*. The gene map was adapted from FlyBase. *Chr* chromosome, *k* kilobase (PDF 38 kb)

## Abbreviations

bp: base pair
kb: kilobase
GFP: green fluorescent protein
OD: optical density of the pellet
PBS: phosphate-buffered saline
PO: phenoloxidase
PPO: prophenoloxidase
qRT-PCR: real-time quantitative reverse transcription PCR
SP: serine protease
