# Supplementary figures and images for: Drosophila innate immunity: regional and functional specialization of prophenoloxidases
Source: BMC Biol. 2015 Oct 1;13:81. doi: 10.1186/s12915-015-0193-6 (PMC4595066; doi:10.1186/s12915-015-0193-6)

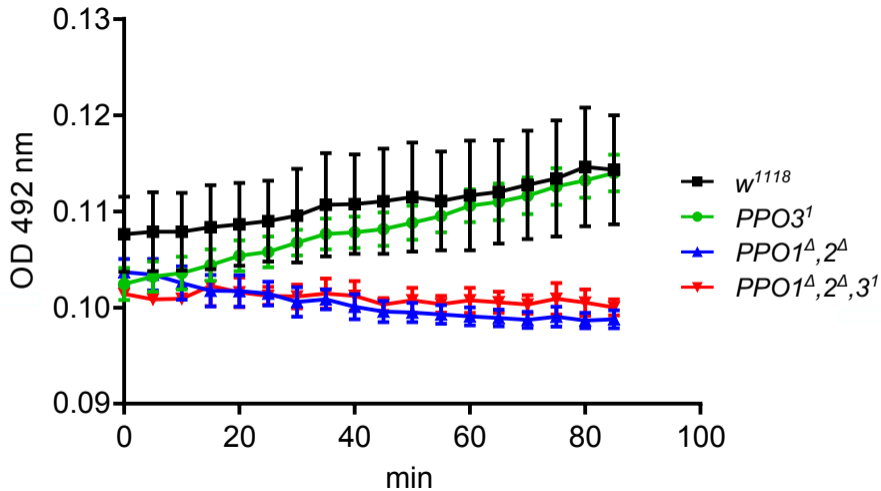

Supplement: Additional file 1: Figure S1. — PPO3 does not contribute to hemolymph PO activity. Hemolymph PO activity of unchallenged larvae was monitored with an L-DOPA assay. Spontaneous PO activity is not affected in PPO3 1 mutant larvae compared to wild-type larvae. The hemolymph of wandering larvae was collected and examined for PO activity by transferring L-DOPA to dopachrome. The hemolymph of PPO1,2,3 mutant larvae was used as a negative control. Differences between PPO3 1 and the w 1118 wild-type control do not reach statistical significance. Data were analyzed by two-way ANOVA with Tukey correction. Values represent the mean ± standard error of the mean of three independent experiments. OD optical density (PDF 20 kb) [file 12915_2015_193_MOESM1_ESM.pdf]

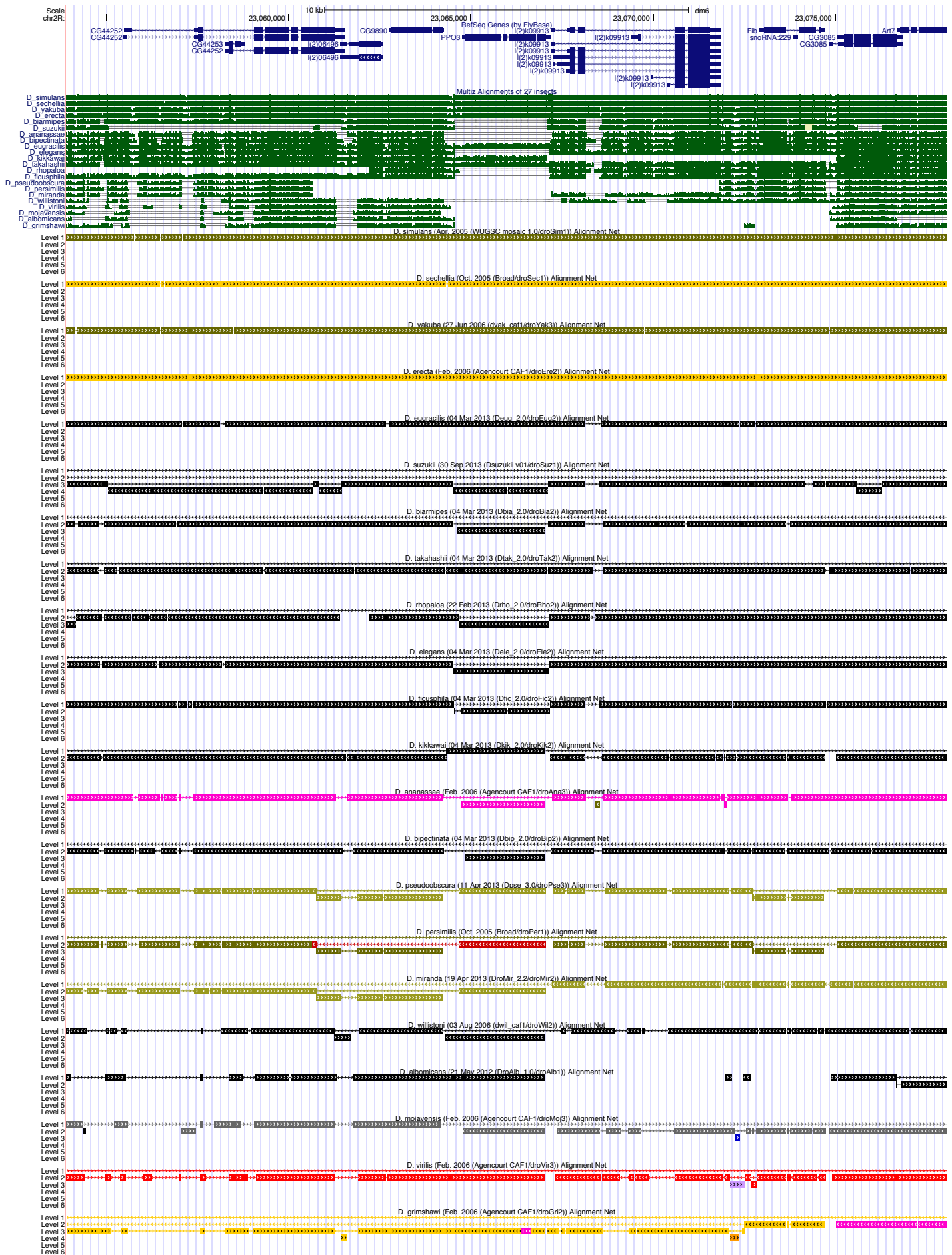

Supplement: Additional file 2: Figure S2. — Comparative genomics of the PPO3 locus. Shown are the best pairwise genome alignments for 22 Drosophila species versus D. melanogaster based on the UCSC genome browser net tracks. Species in the D. melanogaster group plus D. eugracilis have only one net that aligns contiguously the entire PPO3 microsyntenic region. Conversely, other species (besides D. takahashii) have a gap in the alignment at PPO3 that is filled by a secondary net that arises from alignment to paralogous sequences elsewhere in the genome. D. takahashii has only a single alignment net in the PPO3 region; however, this net is fragmented precisely in the PPO3 gene, consistent with the possible presence of non-canonical PPO3-like gene sequences in this species (PDF 1061 kb) [file 12915_2015_193_MOESM2_ESM.pdf]

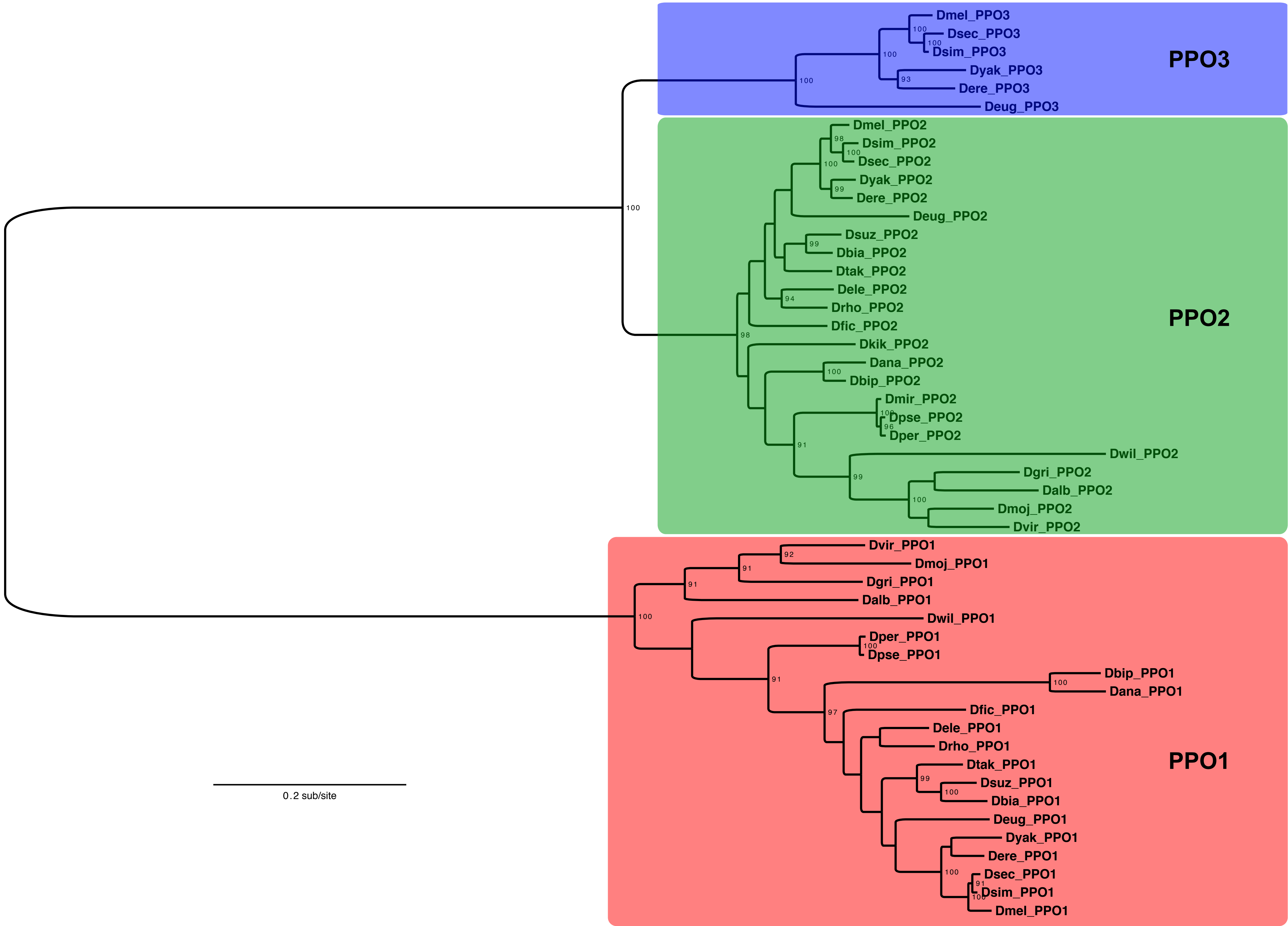

Supplement: Additional file 3: Figure S3. — Phylogenetic tree of PPO genes from Drosophila species. A maximum likelihood phylogenetic tree was constructed with the exonic sequences of PPO1, PPO2 and PPO3 using RAxML. Nodes with more than 90 % bootstrap support are labeled. Branch lengths are in substitutions per site. The tree is midpoint rooted and based on a multiple alignment of all PPO genes from D. melanogaster, D. simulans, D. sechellia, D. yakuba, D. erecta, D. eugracilis, D. suzukii, D. biarmipes, D. takahashii, D. rhopaloa, D. elegans, D. ficusphila, D. kikkawai, D. bipectinata, D. ananassae, D. pseudoobscura, D. persimilis, D. miranda, D. willistoni, D. mojavensis, D. virilis, D. albomicans and D. grimshawi. The placement of the root in our tree is supported by a previous phylogenetic analysis of PPO genes across arthropods [8] (PDF 182 kb) [file 12915_2015_193_MOESM3_ESM.pdf]

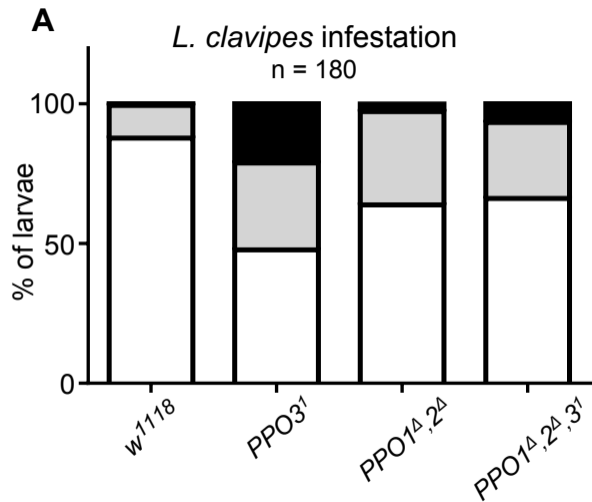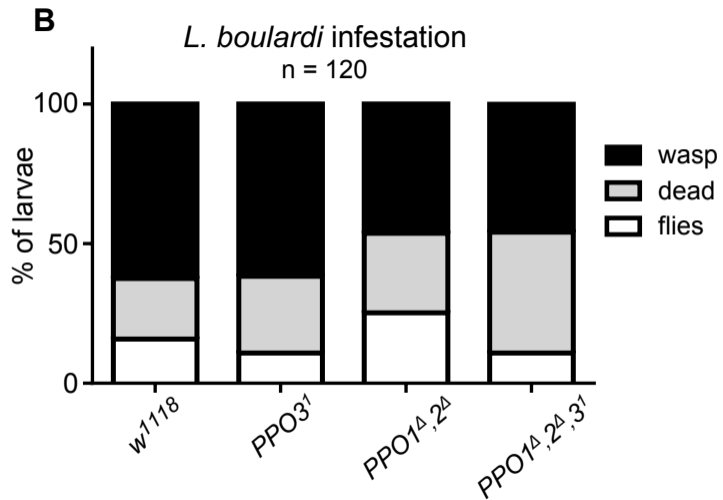

Supplement: Additional file 4: Figure S4. — Survival analysis after Leptopilina clavipes and L. boulardi wasp infestation in wild type and PPO mutants. Synchronized L2 larvae were exposed to gravid female L. clavipes (A) or L. boulardi (B) wasps in custom infection traps, re-isolated and cultured at room temperature until wasps emerged. Vials were scored daily for total number numbers of eclosed flies and wasps. PPO3 mutant larvae show enhanced susceptibility against L. clavipes, whereas the loss of all PPO does not further increase this effect. The mild effect of PPO mutants on wasp encapsulation led us to hypothesize that optimal encapsulation requires a precise level of phenoloxidase activity due to a possible toxic effect on the host (PDF 41 kb) [file 12915_2015_193_MOESM4_ESM.pdf]

**A**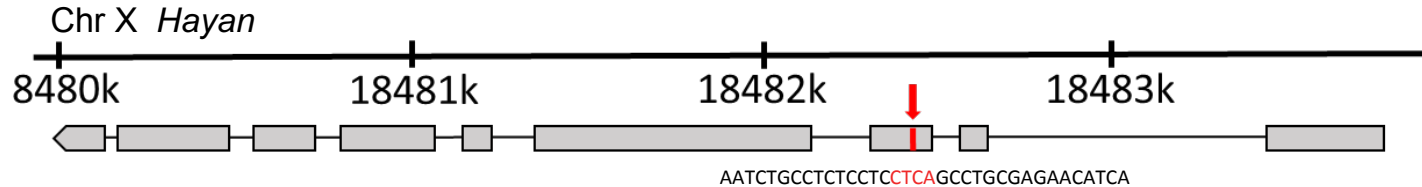**B**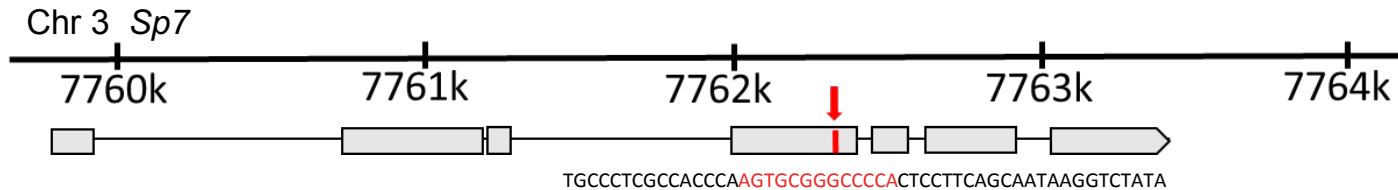

Supplement: Additional file 5: Figure S5. — Schematic representation of novel CRISP/Cas9 mutants. Overview of the Hayan (A) and Sp7 (B) genes. Arrows indicate the position of the mutations. Partial sequences are shown, and deleted nucleotides of Hayan SK3 and Sp7 SK6 are marked in red. The gene map was adapted from FlyBase. Chr chromosome, k kilobase (PDF 38 kb) [file 12915_2015_193_MOESM5_ESM.pdf]
